# CoeViz: A Web-Based Integrative Platform for Interactive Visualization of Large Similarity and Distance Matrices

**DOI:** 10.3390/data3010004

**Published:** 2018-01-13

**Authors:** Frazier N. Baker, Aleksey Porollo

**Affiliations:** 1 Department of Electrical Engineering and Computing Systems, University of Cincinnati, Cincinnati, OH 45221, USA; 2 Center for Autoimmune Genomics and Etiology, Cincinnati Children's Hospital Medical Center, Cincinnati, OH 45229, USA; 3 Division of Biomedical Informatics, Cincinnati Children's Hospital Medical Center, Cincinnati, OH 45229, USA; 4 Department of Pediatrics, University of Cincinnati College of Medicine, Cincinnati, OH 45267, USA

**Keywords:** distance matrix, similarity matrix, interactive visualization, zoomable heatmap, interactive dendrogram, circular relationship diagram, multi-dimensional scaling, CoeViz

## Abstract

Similarity and distance matrices are general data structures that describe reciprocal relationships between the objects within a given dataset. Commonly used methods for representation of these matrices include heatmaps, hierarchical trees, dimensionality reduction, and various types of networks. However, despite a well-developed foundation for the visualization of such representations, the challenge of creating an interactive view that would allow for quick data navigation and interpretation remains largely unaddressed. This problem becomes especially evident for large matrices with hundreds or thousands objects. In this work, we present a web-based platform for the interactive analysis of large (dis-)similarity matrices. It consists of four major interconnected and synchronized components: a zoomable heatmap, interactive hierarchical tree, scalable circular relationship diagram, and 3D multi-dimensional scaling (MDS) scatterplot. We demonstrate the use of the platform for the analysis of amino acid covariance data in proteins as part of our previously developed CoeViz tool. The web-platform enables quick and focused analysis of protein features, such as structural domains and functional sites.

## 1. Introduction

Similarity and distance matrices (SMs and DMs) are common data structures to represent interrelationships within a given set of objects. These matrices can be used for the identification of clusters of the objects, inference of networks and communities, estimation of density of distribution, and other applications requiring quantitative measures of relatedness between the objects. While the field of analyzing and visualizing these matrices is well established, challenges remain for presenting large datasets and providing interactive means for data browsing and analysis.

Heatmaps, dendrograms, circular relationship diagrams, networks, and dimensionality reduction scatterplots are popular methods for visualizing similarity and distance matrices. Heatmaps resemble a grid, with each cell colored according to the distance between a given pair of objects. The colors are normally a gradient of shades to represent the min–max range of all distances in the matrix. The main diagonal can be left blank or contain additional information pertaining to a given single object (e.g., size, weight, or any other individual quantitative property). While such visualization is intuitive and revealing, it is not well suited for large matrices (e.g., with thousands objects), which would be difficult to accommodate simultaneously on a single plot on any medium, such as paper or a computer screen.

Dendrograms are tree diagrams based on a hierarchical clustering derived from the symmetric dissimilarity scores (or distances). Branching points in dendrograms represent distances between the clusters of objects. Dendrograms offer the benefit of reordering the data and grouping together closely related items, which are then easy to recognize visually. The distance axis in a dendrogram is scalable, but the data axis needs to accommodate all data points, which for large datasets is impractical to visualize all at the same time. Navigation through large data is also a challenge.

Circular relationship diagrams are useful for visualizing pairwise relationships in a compact manner. Objects located on circumference are connected by arcs that may represent distances via thickness or color and reveal “hubs”—the most connected objects. However, these diagrams are again limited by the number of objects whose labels can appear in a practical view. In addition, for highly connected data, the connections within the circular diagram become too cumbersome to infer any relationships visually.

Networks are a natural way of displaying the interconnected objects. For example, a Voronoi decomposition (Voronoi diagram) can be uniquely determined by a given DM. However, for complex datasets, its visual representation may be not intuitive. Force-directed graphs (FDGs) are an alternative and more intuitive network representation of DMs. Following the notion of spring-like interactions, FDG balance the attractive and repulsive forces to lay out the nodes representing the objects. These forces can be defined using different functions beyond laws of gravity, charged particles, etc., and may account for specific nature of the objects or their relationships. Again, the main challenge for network representation is to visualize large datasets, which may appear as cumbersome clump of nodes.

Dimensionality reduction can be used to scale distance matrices down to lower dimension coordinates such that they can be rendered on a two- or three-dimensional scatter plot. One such commonly used method is multidimensional scaling (MDS). For large datasets, it may present a challenge to visualize the data, especially when point labels are needed to locate individual objects. An interactive view with zooming capability may facilitate the visual inspection of MDS data.

To address the aforementioned limitations of the existing approaches to visualizing large similarity and distance matrices, we present a web-based platform that combines heatmaps, dendrograms, circular relationship diagrams, and MDS plots into one interactive data visualization tool, with all components synchronized as the user examines the data. To illustrate the data visualization platform, we apply it to the analysis of a covariance matrix for the multi-domain protein, human estrogen receptor-alpha (ESR1), which contains almost six hundred amino acid residues. The presented platform is a further improvement of the previously developed web-server CoeViz [1].

CoeViz can generate a covariance matrix for amino acid residues within a given protein based on multiple sequence alignment (MSA), and using different metrics, including mutual information (MI), chi-squared (χ^2^), and Pearson correlation (r). These matrices are further transformed into distance matrices. Since proteins frequently have long sequences—hundreds or even thousands of residues—there was a need to develop a web-based platform for interactive visualization of such large SMs and DMs to enable an efficient analysis of the covariance data for the identification of couplings of either individual positions or clusters of amino acids that may have shared structural or functional constraints through evolution of a given protein.

## 2. Results

### 2.1. An Integrative Platform for Similarity/Distance Data Visualization

This work represents a major update of CoeViz with regard to the online visualization of covariance matrixes. Specifically, we incorporated an interactive cladogram of the hierarchical clustering tree, which now allows for the manual highlighting of clusters, reacts to the selection of tree leaves by refocusing other visual components to a newly selected residue, and auto-scrolls to a tree leaf when the user selects a residue in other views. We also added a new component—3D MDS scatterplot—that allows the user to view a distance matrix interactively in 3D and identify groupings of residues. All visual components are now synchronized and automatically update all views upon changing focus in one component.

**Figure 1:**
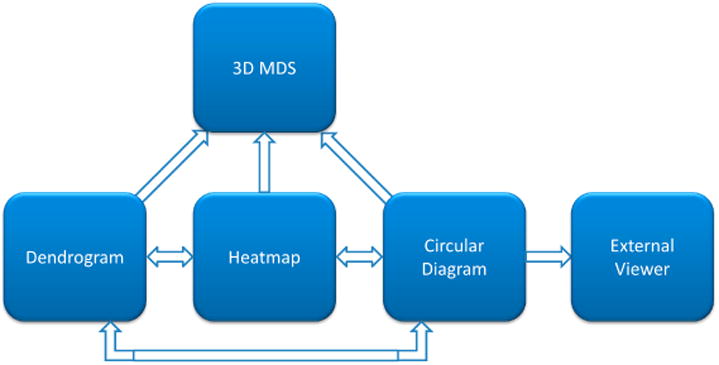
A diagram of the interaction of the visualizations components in CoeViz. Each arrow indicates how the user can navigate through the visualizations from one method to another. One-directional arrows indicate that the viewed data can be updated in the targeted module only upon the change in focus of another module. Two-directional arrows indicate that data visualized is synchronized both ways. External viewers are dedicated to protein sequence/structure view and include POLYVIEW-2D [2], POLYVIEW-3D [3], and Jmol [4].

The developed web-based platform for interactive visualization of similarity and distance matrices consists of four major interconnected components: heatmap, dendrogram, circular diagram, and three-dimensional MDS scatterplot. Figure 1 illustrates interaction of these components in CoeViz. Their specific implementation is described below.

**Figure 2:**
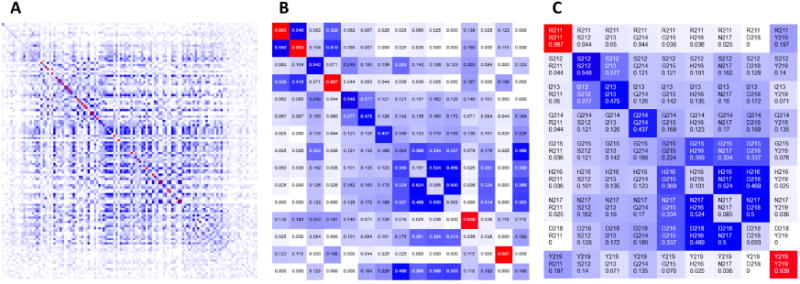
Implementation of the interactive heatmap visualization of covariance metrics in CoeViz. The heatmap can display (**A**) an entire domain, (**B**) covariance scores, and (**C**) labels of co-varying residues.

The heatmap presents covariance data (or similarity data, in general) based on one of the implemented covariance metrics [1]. The color gradient spans from white (representing no covariance, 0), through blue (moderate, 0.5) to red (high covariance, 1). The main diagonal contains frequencies of given amino acids observed at the individual positions in a given MSA. Heatmaps are zoomable from single pixel per position to large grid cells presenting detailed information, such as row and column indexes, corresponding residue labels and covariance scores. For quick navigation, heatmaps can be dragged with a mouse to pan to another part of the grid or be refocused using either a navigation pane or another visualization component. Figure 2 shows the same heatmap at different zoom levels.

**Figure 3:**
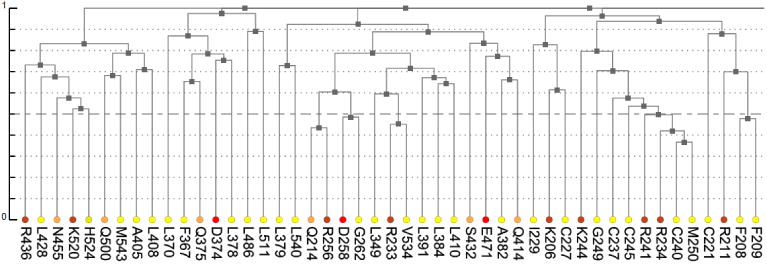
A fragment of the dendrogram derived from hierarchical clustering of co-varying residues (leaves). Colors reflect physico-chemical properties of amino acids. The color notation is as previously defined [2].

The dendrogram presents results of hierarchical clustering of covariance data transformed into a distance matrix. In the context of protein data, leaves of the tree dendrogram are colored according to physico-chemical properties of amino acids (Figure 3). The added interactivity of the dendrogram greatly improves navigation through the data and synchronization of visualization. When a leaf in the dendrogram is clicked, it highlights the cell in the main diagonal of the heatmap and opens (or refocuses) the circular relationship diagram for that residue. To account for large proteins, the tree view is scrollable and automatically refocuses on a residue when it is chosen by the user in another interactive visualization component.

**Figure 4:**
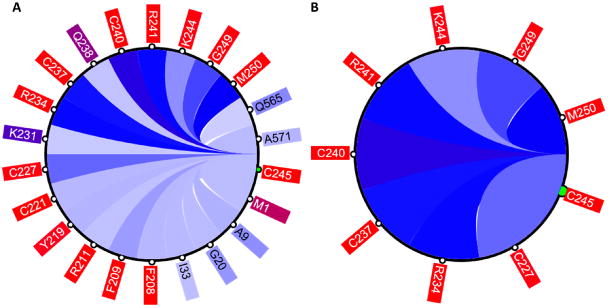
The circular relation diagram centered on the residue C245 with its top co-varying residues at cutoffs (**A**) 0.1 and (**B**) 0.2. Labels on the diagram represent amino acid residues and their positions in protein sequence. Colors of these labels are the same as those on the main diagonal of the heatmap. Colors of the arcs represent covariance scores between two given positions.

The circular relation diagram (CD) is automatically updated for each newly chosen residue and, by default, displays top 5% of the most co-varying residues with the chosen residue. The number of residues shown can be altered by changing the cutoff of covariance scores (Figure 4). The diagram can be interactively expanded to show the same data in the table format. One can refocus the view to any residue in the diagram to reveal its own set of the top co-varying residues. Such refocus invokes an instant update of the three other visual components to reflect the change in focus. The CD also enables the external visualization of the residues displayed in the diagram using the POLYVIEW web-based platform: POLYVIEW-2D [2], POLYVIEW-3D [3], and Jmol [4]. The latter two options are available only when a protein 3D structure was used as an input for CoeViz analysis. The Jmol view enables the interactive analysis of the structural arrangement of the selected co-varying residues facilitating the inference of their structural and/or functional relationships.

**Figure 5:**
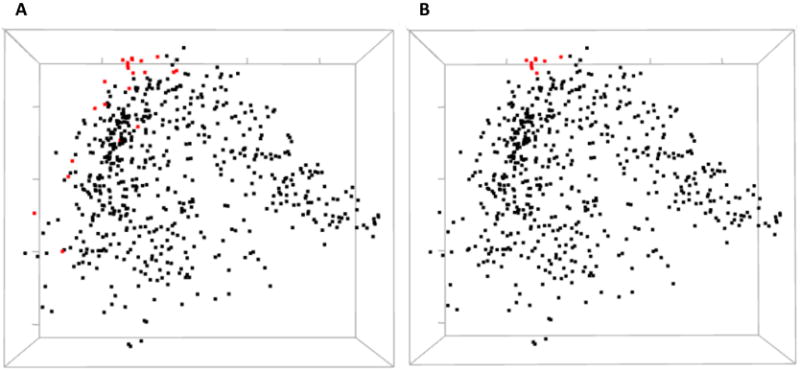
3D multidimensional scaling (MDS) scatterplots of co-varying residues in human ESR1. (**A**) Highlighted red are the residues corresponding to panel **A** of Figure 4. (**B**) Red dots correspond to residues on panel **B** of Figure 4. Both black and red dots can be optionally labeled with the residue identifiers but not shown here for a more clear view.

Three-dimensional view of MDS allows for a global yet compact presentation of relationships between the residues (Figure 5). From covariance data projected into 3D by MDS, one can identify domains of the protein, some small clusters of functionally relevant residues, and residues standing away from the rest. The 3D view pane provides interactive zoom-in and rotation capabilities, as well as the labeling of selected residues. Current implementation of the MDS view does not allow for the interactive selection of individual residues on the scatterplot to be used for refocusing views in other CoeViz components due to limitations of the R library used.

### 2.2. Analysis of Human ESR1

Human estrogen receptor alpha (ESR1) is a multi-domain protein that belongs to the family of nuclear receptors. It represents an interesting object for the amino acid covariance analysis and visualization since its domains, while all serve the purpose of a transcription factor, play distinct molecular functions detailed below. The domains also contain additional functional regions, such as zinc coordinating residues (Zinc fingers) in the DNA-binding domain and ligand binding residues in the transactivation domain AF2.

**Figure 6:**
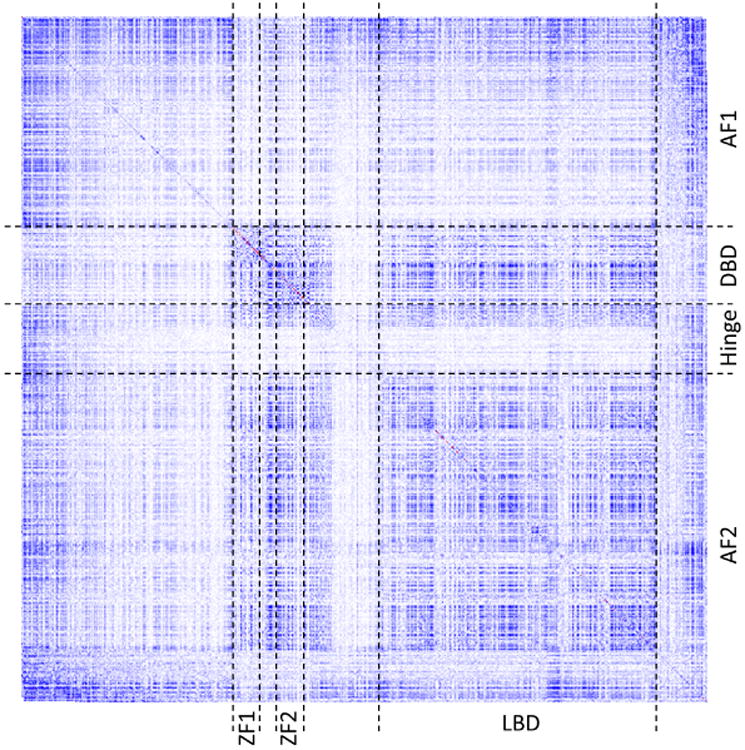
The heatmap of co-varying residues in human ESR1 protein as measured using χ2 statistic adjusted for phylogenetic bias in the multiple sequence alignment. White points indicate no covariance, blue—moderate, and red—high covariance between a pair of residues. Points on the main diagonal represent frequencies of observing a given amino acid throughout the MSA at a given position. Boundaries of the domains and regions were marked with black dashed lines following the annotation of the UniProt database. Domains: AF1—transactivation domain 1; DBD—DNA-binding domain; Hinge – hinge domain; AF2—transactivation domain 2. Functional regions: ZF1 and ZF2— zinc finger domains; LBD—ligand binding domain.

Full protein sequence of ESR1 (595 amino acids) was submitted for the analysis by CoeViz using the χ^2^ covariance metric adjusted for phylogenetic bias in the MSA. Figure 6 shows a heatmap of covariance scores for residues across the entire protein. As can be seen from the figure, the boundaries of the patterns of co-varying residues by and large coincide with the known domains and functional regions of the protein.

We further interrogated as to whether residues involved in distinct functions, such as metal coordination, DNA- and ligand-binding, or those involved in protein–protein interaction can be identified as separate clusters or what other residues they are clustered with.

**Figure 7:**
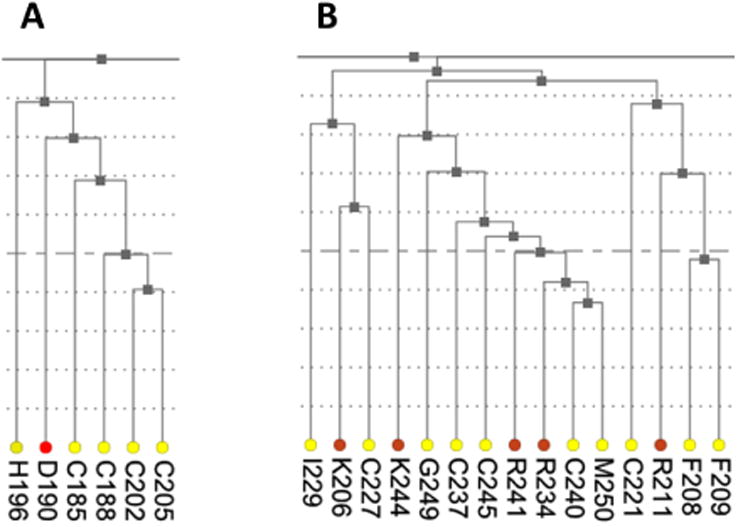
Fragments of the hierarchical clustering tree for co-varying residues in human ESR1. (**A**) Cluster containing the metal coordinating residues from Zn finger 1 (C185, C188, C202, and C205). (**B**) Cluster containing the metal coordinating residues from Zn finger 2 (C221, C227, C237, and C240).

As was mentioned earlier, ESR1 comprises two Zn fingers in its DNA-binding domain. From each Zn finger, we picked the first residue that is known to coordinate a Zn^2+^ ion: C185 and C221 from ZF1 and ZF2, respectively. Figure 7 shows that these residues were clustered with their partners, metal coordinating residues C188, C202, and C205 and C227, C237, and C240, respectively. The same two clusters also contain residues directly binding DNA: H196, K206, R211, R234, and R241. Other DNA binding residues—Y195, Y197, E203, G204, A207, K210, K235, and Q238—did not form a distinct cluster.

**Figure 8:**
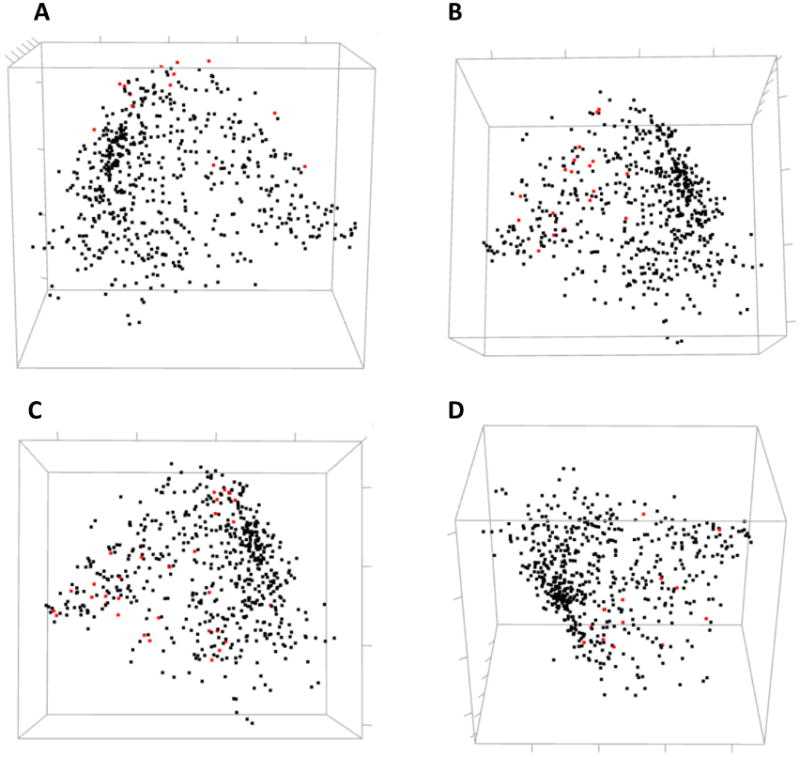
Three-dimensional MDS scatterplots of co-varying amino acids in human ESR1. Highlighted in red are the residues involved in different molecular functions: (**A**) DNA binding; (**B**) binding estradiol; (**C**) homodimerization of hormone receptor; (**D**) interaction with a co-activator.

Residues involved in direct ligand (estradiol) binding or in protein dimerization and interaction with a co-activator were not clustered together by hierarchical clustering. Still, one can analyze their mutual covariance-based distances using an interactive 3D MDS scatterplot (Figure 8).

### 2.3. Comparison with Other Existing Tools

The presented tool is meant to illustrate the general concept of the visualization of large (dis-)similarity matrixes via synchronized orthogonal views. However, since the examples presented here pertain to the covariance data in proteins, a number of existing servers for coevolution analysis in proteins were evaluated. Based on the original publications, where some visualization means for the results were presented, we tried ConEVA [5], EVcouplings [6], and GREMLIN [7] using the same human ESR1 protein.

**Figure 9:**
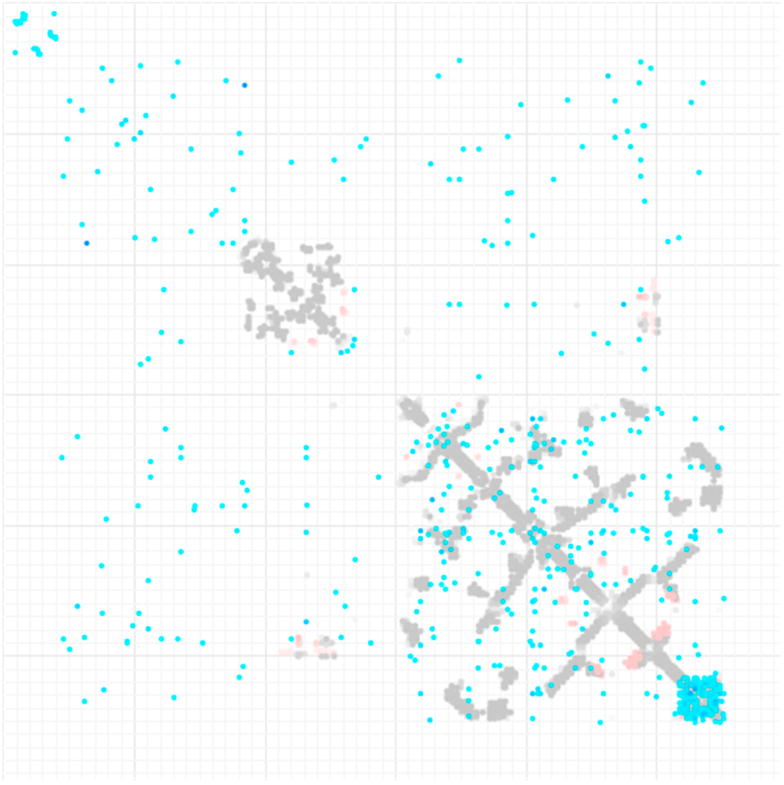
Results of the GREMLIN server [7] for human ESR1 overlaid with known residue contacts found in Protein Databank (PDB). Blue filled circles are GREMLIN results (scaled score > 1). The grey/red filled circles underneath are PDB residue contacts (minimal distance < 5 Å). The shade of the circles is based on 10 HHsearch results. Inter-oligomeric contacts in the PDB are in shades of red.

The ConEVA web-server was not responsive after multiple attempts, so it may be no longer supported. EVcouplings accepted the protein input with the remaining parameters used as defaults. No results were returned after two days post submission. It is possible that the server is not meant for large or multi-domain proteins. GREMLIN accepted the input with the warning “Note, due to limited resources, your submission may take forever to complete (Jobs Running: 0).” Nevertheless, the server found identical query protein submitted previously by another user and returned results with the input parameters used as specified by that user. Figure 9 contains the output provided by GREMLIN, where covariance analysis is overlaid with the pairwise residue contact information collected through the Protein Databank entries containing homologous protein chains.

The contact map for ESR1 from GREMLIN is static, with no interactive functionality or mouse hover information provided, which makes it difficult to locate what pair of residues a given pixel/shade represents. It should be noted that GREMLIN does provide an interactive analysis for generated covariance data when a 3D structure is available for a given protein sequence. Collectively, other existing servers either do not provide as versatile visualization techniques as CoeViz does or are not capable of processing large and/or multi-domain proteins in a reasonable time frame.

## 3. Discussion

Similarity or distance matrices are a natural way of presenting relationships between objects. However, analysis and visualization of such matrices for large datasets remain challenging. Different clustering algorithms and visualization methods usually have various strengths and weaknesses. To improve the process of visualization and navigation through the data, we have implemented an online platform for interactive visualization that combines a zoomable heatmap, an auto-scrolling hierarchical clustering tree, a scalable circular relationship diagram, and an interactive 3D multidimensional scaling scatterplot. All components are interconnected and synchronized, which greatly facilitates the large data analysis.

The purpose of this work is to demonstrate the concept of interactive multi-faceted analysis of large SMs and DMs. The analysis of covariance data in proteins was used as an illustration of the platform utility; when using the different approaches combined, one could easily browse the data and infer related objects from the sparse, noisy data. None of the individual methods alone would allow for such efficient data navigation and analysis.

## 4. Materials and Methods

### 4.1. Web Implementation

The client side of the CoeViz interface is based on JavaScript libraries, including D3 and WebGL. The server side runs on Perl, Python, and R scripts.

The heatmap and circular diagram were implemented using the D3 library [8]. D3 is used for manipulating the document object model (DOM), processing the data, providing interactivity, and efficient rendering the graphics on the HTML canvas.

For the dendrogram, a JSON file from the output of the R hclust function is generated using the jsonlite library [9]. Residues are clustered using the complete linkage hierarchical clustering algorithm. The JSON file is then loaded into the CoeViz web page to render an interactive dendrogram with animations using SVG elements.

The MDS scatterplot is generated using the RGL R library [10]. The R cmdscale function reduces the distance matrix to three dimensions and then RGL generates a WebGL code for the interactive HTML visualization.

Heatmaps and MDS plots can be exported as images in PNG format, whereas circular diagrams and dendrograms are exported in SVG format.

The CoeViz web application is available via http://polyview.cchmc.org/. Documentation with interactive examples can be found at http://polyview.cchmc.org/coeviz_doc.html. The JavaScript and R code for the integrated web application is available from http://github.com/frazierbaker/coeviz. The interactive dendrogram component is available standalone at http://github.com/frazierbaker/d3ndro or as an NPM package under the name “d3ndro.”

Details on computing MSA and covariance scores can be found in the original CoeViz publication [1] as well as in the documentation web-page specified above.

### 4.2. Annotation of Protein Structure and Function

Protein sequence of human ESR1 has been retrieved from the UniProt database (ID: P03372). The same UniProt entry was used to retrieve information about boundaries of structural domains and functional regions. Resolved parts of the protein structure were retrieved from the Protein Databank [11]: PDB ID 1hcq—DNA-binding domain; PDB ID 3uud—ligand binding domain co-crystallized with its natural ligand estradiol and protein interaction partners. The following tools were used to retrieve additional information about specific residues based on the resolved structures: POLYVIEW-2D [2] for the identification of metal and DNA binding residues and SPPIDER [12] for the analysis of protein–protein interaction sites.

## References

[1] Baker FN, Porollo A (2016). CoeViz: A web-based tool for coevolution analysis of protein residues. BMC Bioinform.

[2] Porollo AA, Adamczak R, Meller J (2004). Polyview: A flexible visualization tool for structural and functional annotations of proteins. Bioinformatics.

[3] Porollo A, Meller J (2007). Versatile annotation and publication quality visualization of protein complexes using polyview-3D. BMC Bioinform.

[4] Hanson RM (2010). Jmol—A paradigm shift in crystallographic visualization. J Appl Crystallogr.

[5] Adhikari B, Nowotny J, Bhattacharya D, Hou J, Cheng J (2016). Coneva: A toolbox for comprehensive assessment of protein contacts. BMC Bioinform.

[6] Marks DS, Hopf TA, Sander C (2012). Protein structure prediction from sequence variation. Nat Biotechnol.

[7] Ovchinnikov S, Kamisetty H, Baker D (2014). Robust and accurate prediction of residue-residue interactions across protein interfaces using evolutionary information. Elife.

[8] Bostock M, Ogievetsky V, Heer J (2011). D-3: Data-driven documents. IEEE Trans Vis Comput Graph.

[9] Ooms J, Temple Lang D, Hilaiel L Jsonlite: A Robust, High Performance JSON Parser and Generator for R. https://cran.r-project.org/web/packages/jsonlite/index.html.

[10] Adler D, Nenadic O, Zucchini W (2003). RGL: A R-library for 3D visualization with OpenGL.

[11] Berman HM, Westbrook J, Feng Z, Gilliland G, Bhat TN, Weissig H, Shindyalov IN, Bourne PE (2000). The protein data bank. Nucleic Acids Res.

[12] Porollo A, Meller J (2007). Prediction-based fingerprints of protein-protein interactions. Proteins.

